# The Human WBSCR22 Protein Is Involved in the Biogenesis of the 40S Ribosomal Subunits in Mammalian Cells

**DOI:** 10.1371/journal.pone.0075686

**Published:** 2013-09-23

**Authors:** Kadri Õunap, Ly Käsper, Ants Kurg, Reet Kurg

**Affiliations:** 1 Center of Biomedical Technology, Institute of Technology, University of Tartu, Tartu, Estonia; 2 Institute of Molecular and Cell Biology, University of Tartu, Tartu, Estonia; University of Lethbridge, Canada

## Abstract

The human WBSCR22 protein was previously shown to be up-regulated in invasive breast cancer and its ectopic expression enhances tumor cell survival in the vasculature. In the current study, we show that the WBSCR22 protein is important for cell growth. Knock-down of WBSCR22 with siRNA results in slower growth of WBSCR22-depleted cells. Treatment with siWBSCR22 causes defects in the processing of pre-rRNAs and reduces the level of free 40S ribosomal subunit, suggesting that WBSCR22 is involved in ribosome small subunit biosynthesis. The human WBSCR22 partially complements the growth of WBSCR22 yeast homologue, *bud23* deletion mutant suggesting that the human WBSCR22 is a functional homologue of yeast Bud23. WBSCR22 is localized throughout the cell nucleus and is not stably associated with ribosomal subunits within the cell nucleus. We also show that the WBSCR22 protein level is decreased in lymphoblastoid cell lines derived from William-Beuren Syndrome (WBS) patients compared to healthy controls. Our data suggest that the WBSCR22 protein is a ribosome biogenesis factor involved in the biosynthesis of 40S ribosomal particles in mammalian cells.

## Introduction

The human methyltransferasome consists of more than 200 proteins making up about 0.9% of all human gene products [1]. Methyltransferases can use a variety of different substrates, including RNA, DNA, small molecules and proteins, and are involved in different biological pathways. They have been shown to be essential in epigenetic control, biosynthesis, protein repair, hormone inactivation, and nucleic acid processing [2,3]. The function and physiological role of many human methyltransferases is still not known. Some methyltransferases characterized so far are associated with disorders, most frequently with cancer and mental disorders [1].

The WBSCR22 protein contains an S-adenosylmethionine (SAM) binding motif typical of seven-β-strand or Rossmann-fold methyltransferases. Recent works have shown that the WBSCR22 protein is expressed at a high level in invasive breast cancer and its ectopic expression enhances tumor cell survival in the vasculature. Knock-down of endogenous WBSCR22 in tumour cells reduced metastasis formation in mouse model. Nakazawa et al. showed that WBSCR22, called Merm1 (metastasis-related methyltransferase 1) in their work, suppressed Zac1 expression by histone H3K9 methylation, and suggested that WBSCR22 might be a histone methyltransferase [4]. In another study, WBSCR22 mRNA was shown to be highly expressed in multiple myeloma cells and regulate the survival of these cells [5].

The human WBSCR22 gene is located in Williams-Beuren Syndrome (WBS) critical region in chromosome 7q11, 23. WBS is a multisystem developmental disorder associated with hemizygous deletion of a ~1.6 Mb region in the given locus. WBS patients display multiple clinical symptoms including cardiovascular diseases, connective tissue abnormalities, intellectual disability (usually mild), growth and endocrine abnormalities [6,7]. The WBS region contains more than 25 genes and the deletion of this region results in haploinsufficiency of WBS control region transcripts [8].

A lot of human methyltransferases have an orthologous partner in yeast. The yeast homologue of WBSCR22, Bud23, sharing 47% of similarity on amino acid level, is a ribosomal 18S rRNA methyltransferase required for ribosome biogenesis [9,10]. Bud23 is a non-essential protein which deletion in yeast results in slow growth phenotype and defects in rRNA processing [9]. Production of ribosomes is a fundamental process that occurs in all dividing cells. Besides ribosomal proteins and rRNAs, more than 150 trans-acting factors, including ribonucleases, RNA helicases, kinases, NTPases and methyltransferases, are required for ribosome biogenesis. Generally, these trans-acting factors are well conserved from yeast to human cells and have similar functions [11,12,13]. The Bud23 homologue in plant *Arabidopsis* , the RID2 protein, is required for cell proliferation and is involved in rRNA processing and nucleolar activity, suggesting for evolutionarily conserved function of the proteins [14].

In the current study, we demonstrate that the human WBSCR22 protein is involved in ribosome biogenesis. Knock-down of WBSCR22 with siRNA slows down the rate of cell growth and leads to decreased amounts of 40S ribosomal subunits relative to 60S subunits. The human WBSCR22 partially complements the growth of *bud23* deletion mutant suggesting that the human WBSCR22 is a functional homologue of yeast Bud23. Our data suggest that these two proteins have similar, but probably not identical functions in ribosome biosynthesis.

## Results

### Depletion of WBSCR22 suppresses cell growth

**Figure 1:**
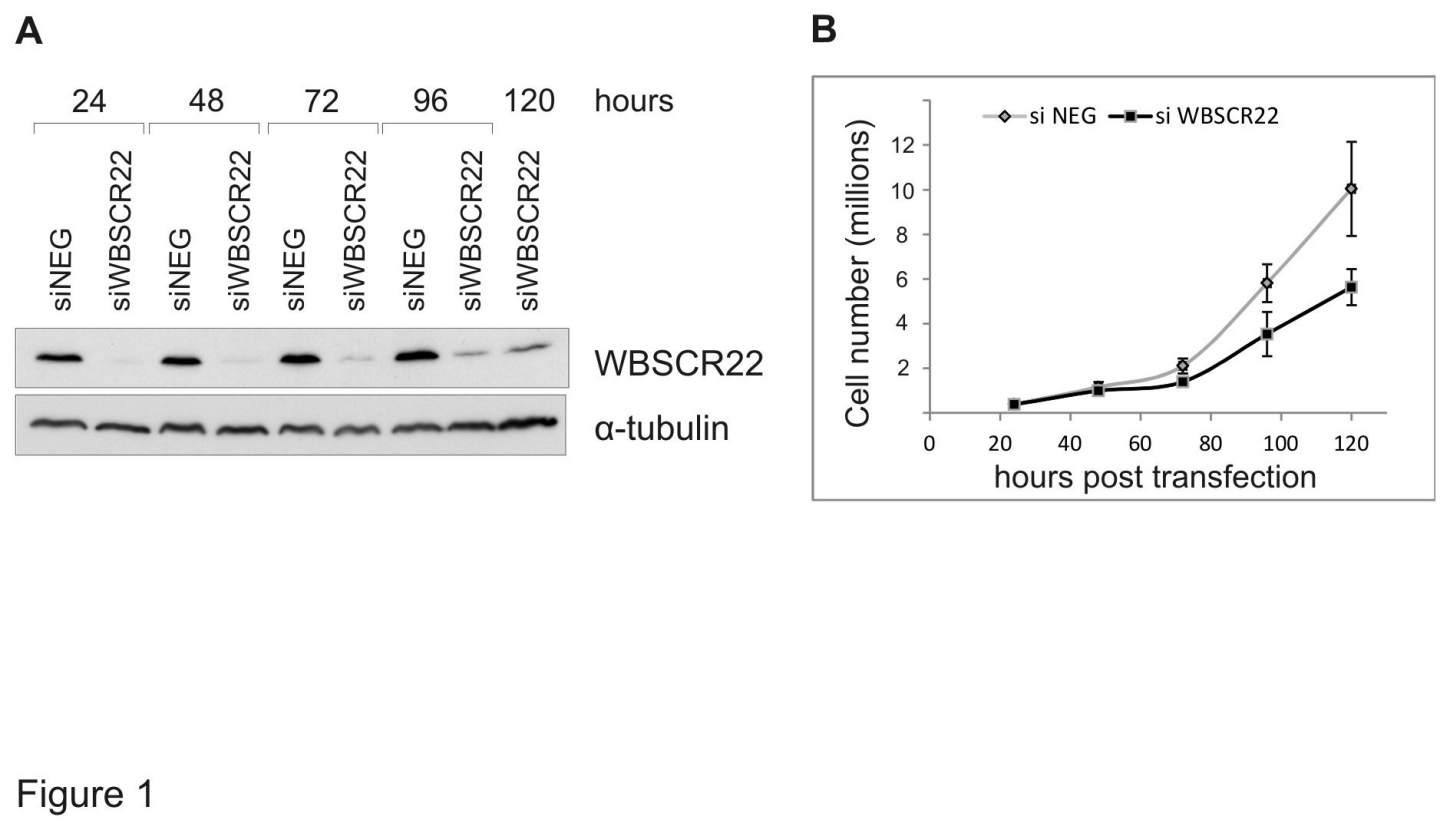
Depletion of WBSCR22 reduces cell growth. (A) Protein expression of siWBSCR22 and siNeg. transfected cells was determined by western blot analysis using anti-WBSCR22 and anti-tubulin antibodies. Proteins from 10^5^ cells are loaded on each lane. (B) HeLa cells were transfected with siWBSCR22 or a control, siNeg, and the cell growth was monitored for five days. Average of three independent transfection experiments is shown.

Recent studies have shown that WBSCR22 is upregulated in some cancer cells, including breast cancer and multiple myeloma cells [4,5]. To investigate the physiological role of WBSCR22 in cell growth, we have knocked down the WBSCR22 protein expression by siRNA. The HeLa cells were electroporated with control and WBSCR22 siRNAs (Figure 1A), and the number of cells was counted up to 120 hours post transfection. As shown in Figure 1B, the number of cells transfected with siRNA specific to WBSCR22 was decreased at 72, 96 and 120 hours post transfection compared to control cells. We calculated the doubling time of WBSCR22-depleted HeLa cells and our data show that the doubling time of HeLa cells transfected with siWBSCR22 was 25 hours instead of the 21 hours for cells transfected with siNeg. Thus, the WBSCR22-depleted cells grow slower than control cells, suggesting that the WBSCR22 protein is important for cell growth.

### WBSCR22 is involved in ribosome biogenesis and rRNA processing

**Figure 2:**
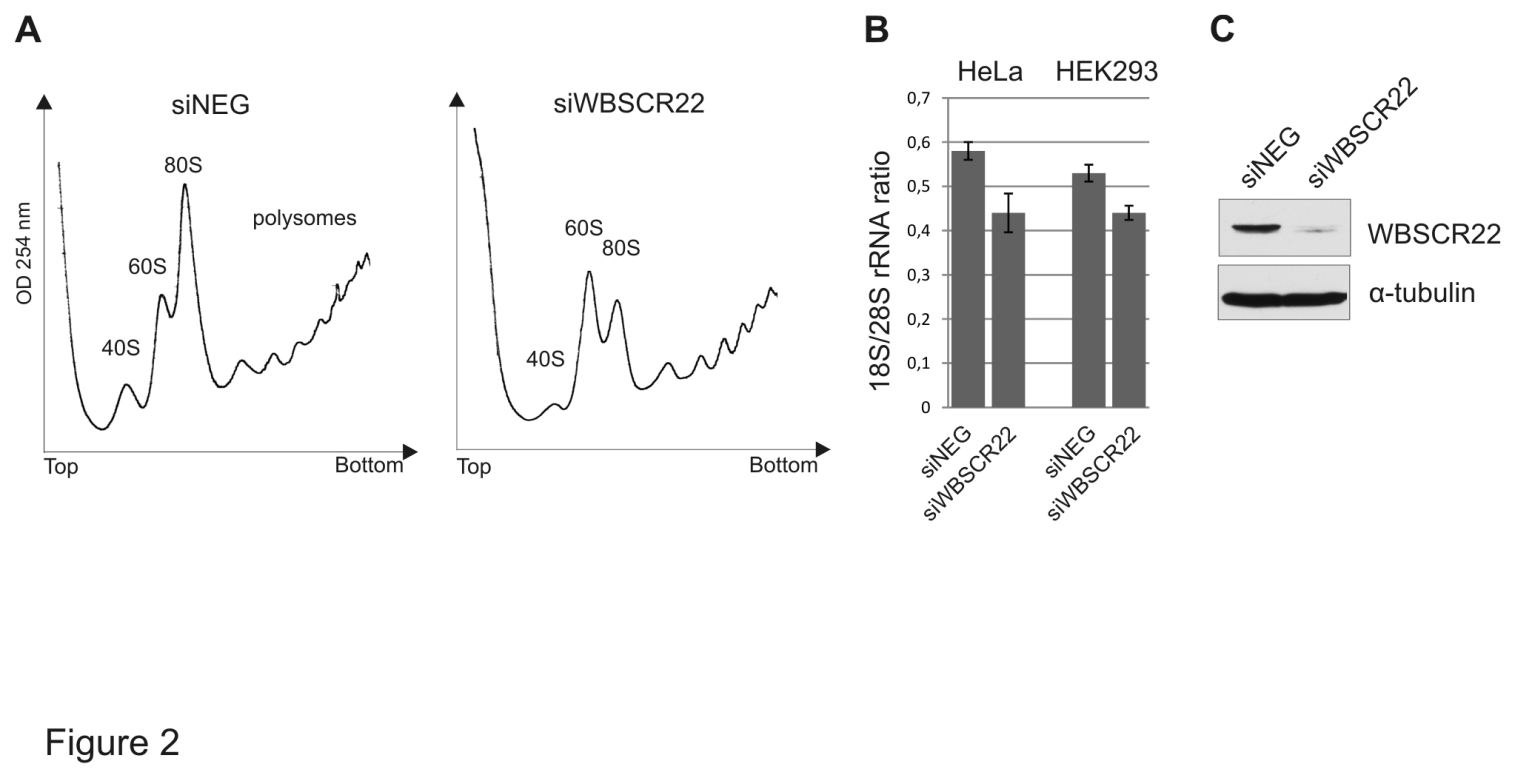
Polysome analysis of HeLa cells after depletion of WBSCR22. (A) HeLa cells were transfected with siWBSCR22 and siNeg, the cytoplasmic cell extracts were prepared 72 h after transfection and centrifuged on 10-50% sucrose gradient. Absorbance at 254 nm was measured across the gradient, and the positions corresponding to the 40S, 60S and 80S ribosomal particles are indicated. (B) The ratio of 18S/28S rRNA from HeLa and HEK293 cells transfected with siRNAs. RNA was purified from cytoplasmic extracts of siWBSCR22 and siNeg cells, separated by electrophoresis and intensities of 18S and 28S rRNA were quantified. The P value is 0.04 using Student’s t-test. (C) Protein expression of siWBSCR22 and siNeg. transfected cells was determined by western blot analysis using anti-WBSCR22 and anti-tubulin antibodies.

The human WBSCR22 protein shares 47% of similarity with its yeast homologue, Bud23, which is a non-essential non-ribosomal protein involved in ribosome biosynthesis [9,10]. Generally, proteins involved in ribosome biosynthesis are well conserved from yeast to human and have similar functions. To study the function of the WBSCR22 protein in ribosome synthesis, HeLa cells were transfected with siWBSCR22 and siNeg, and the ribosome profile of cell extracts prepared in the presence of cycloheximide, which stabilizes polysomes, was analyzed on sucrose density gradients. As shown in Figure 2A, analysis of cytoplasmic extracts showed differences in the ratio of free 40S and 60S subunits between the control and WBSCR22-depleted cells. The levels of free 40S subunits and 80S particles have decreased, and free 60S subunits accumulated in WBSCR22-depleted cells compared to control cells (Figure 2A) referring to defects in 40S subunit synthesis. Depletion of WBSCR22 did not alter either the profile or amount of polysomes 72 hours after transfection. The knock-down of WBSCR22 reduced the amount of mature 18S rRNA in the cytoplasm of the cells (Figure 2B). The level of the WBSCR22 protein after treatment of cells with siRNA is shown in Figure 2C.

**Figure 3:**
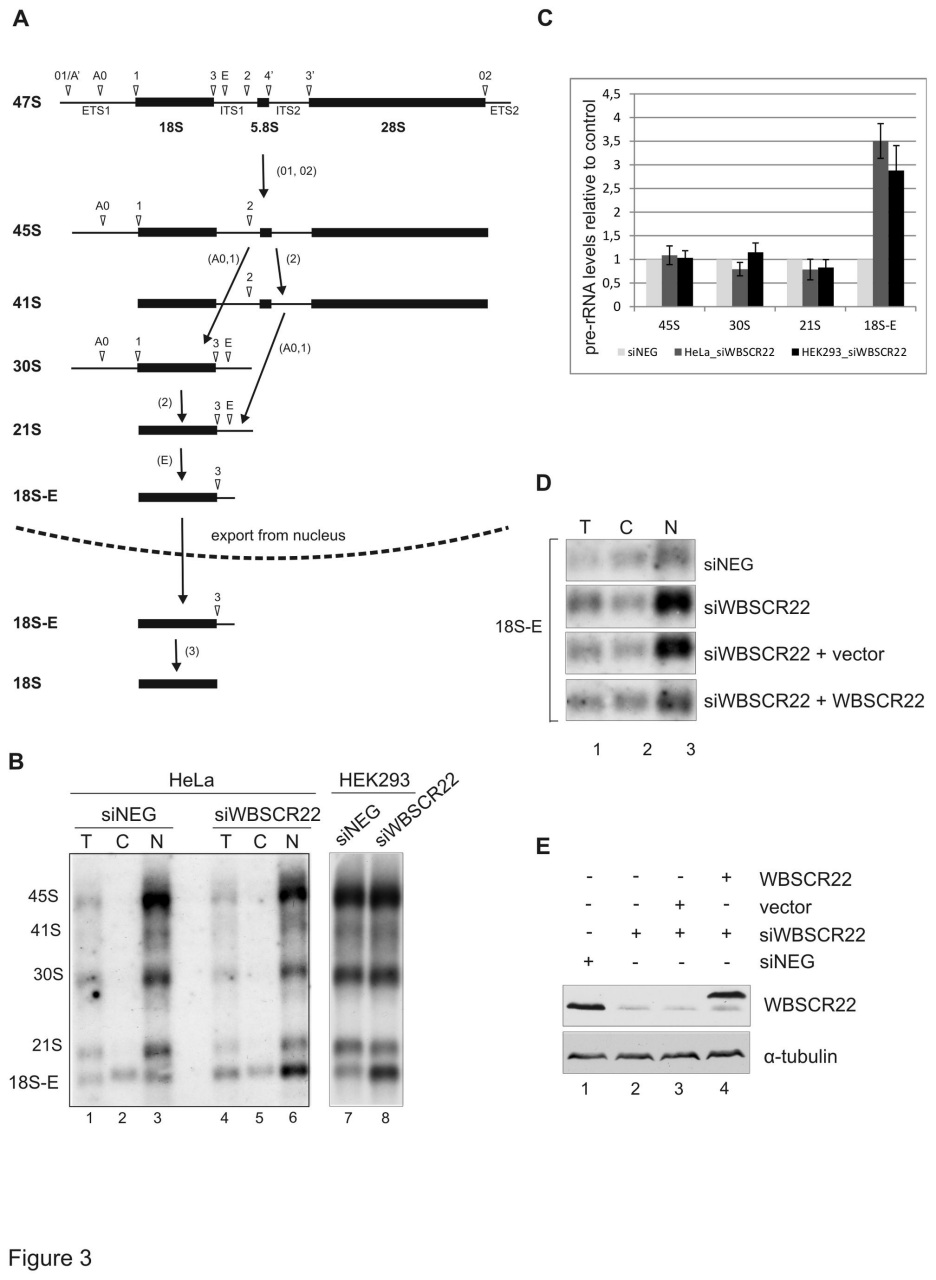
Analysis of pre-rRNA processing in WBSCR22-depleted cells. (A) Pre-rRNA processing in HeLa cells according to Carron et al. [19]. (B) Northern blot analysis of HeLa (lanes 1-6) and HEK293 (lanes 7, 8) cells transfected with siWBSCR22 or siNeg. with the 5’-ITS1 probe. RNA was extracted 72 h after transfection and 3 µg of total (T), 6 µg of cytoplasmic (C) and 3 µg of nuclear (N) RNA were loaded on each lane. Lanes 7 and 8 show nuclear RNA. The positions of precursor rRNAs are indicated. (C) The amounts of pre-rRNAs in the nuclear fractions of HeLa and HEK293 cells were quantified by PhosphorImager and are represented relative to siNeg of appropriate cell line which was set as 1. Results shown are representative of three independent transfection experiments with standard deviations. The P value is 0.2 for 21S and 0.02 for 18S-E using Student’s t-test. (D) Northern blot analysis of rRNA from T (total), C (cytoplasmic) and N (nuclear) fractions with the 5’-ITS1 probe from HeLa cells transfected with siWBSCR22 and plasmids expressing E2Tag-WBSCR22 or epitope tag alone. Band corresponding to 18S-E pre-rRNA is shown. (E) Protein expression of siWBSCR22-depleted cells transfected with expression plasmid for E2Tag-WBSCR22 was determined by western blotting using anti-WBSCR22 and anti-tubulin antibodies.

Next we examined the rRNA processing of 18S rRNA precursors and intermediates by Northern blotting using probe hybridizing to the 5’ of ITS1 linker [12]. Processing of the pre-rRNAs into mature 18S takes place mostly in the nucleus of the cell, however, the final cleavage at the 3’ end of the 18S rRNA and the maturation of 40S particles occurs in the cytoplasm (Figure 3A). In mammalian cells, the pre-40S particles exiting the nucleus contain the 18S-E pre-rRNA, which is the precursor of mature 18S rRNA [12]. Northern blot analysis of whole cell extracts showed slightly increased level of the 18S-E species in WBSCR22-depleted cells (Figure 3B, lanes 1 and 4). Further analysis of nuclear and cytoplasmic RNA revealed that the level of 18S-E pre-rRNA was significantly increased and accumulated in the nucleus of WBSCR22-depleted cells (Figure 3B, compare lanes 3 and 6, 7 and 8), while cytoplasmic fractions of the 18S-E pre-rRNA were comparable between siWBSCR22 and siNeg.-transfected cells (Figure 3B, lanes 2 and 5). Quantification of the Northern blot results showed approximately 3-fold increase of 18S-E signal in the nucleus of both human cell lines, HeLa and HEK293, used in our study (Figure 3C). In addition, slight decrease in the level of 21S was detected in the nucleus of the cell (Figure 3B, C); however, this difference was not statistically significant. In rescue experiments with epitope-tagged WBSCR22 protein, accumulation of 18S-E pre-rRNA was no longer detected in HeLa cells treated with siWBSCR22 (Figure 3D). The levels of WBSCR22 protein in this experiment are shown in Figure 3E. Thus, the physical presence of WBSCR22 protein is required for normal processing of 18S pre-rRNAs. Taken together, our data suggest that the knock-down of WBSCR22 causes defects in the processing of 18S pre-rRNA, suggesting that the WBSCR22 protein is involved in the synthesis of 40S subunits.

### WBSCR22 protein is the functional homologue of yeast Bud23

**Figure 4:**
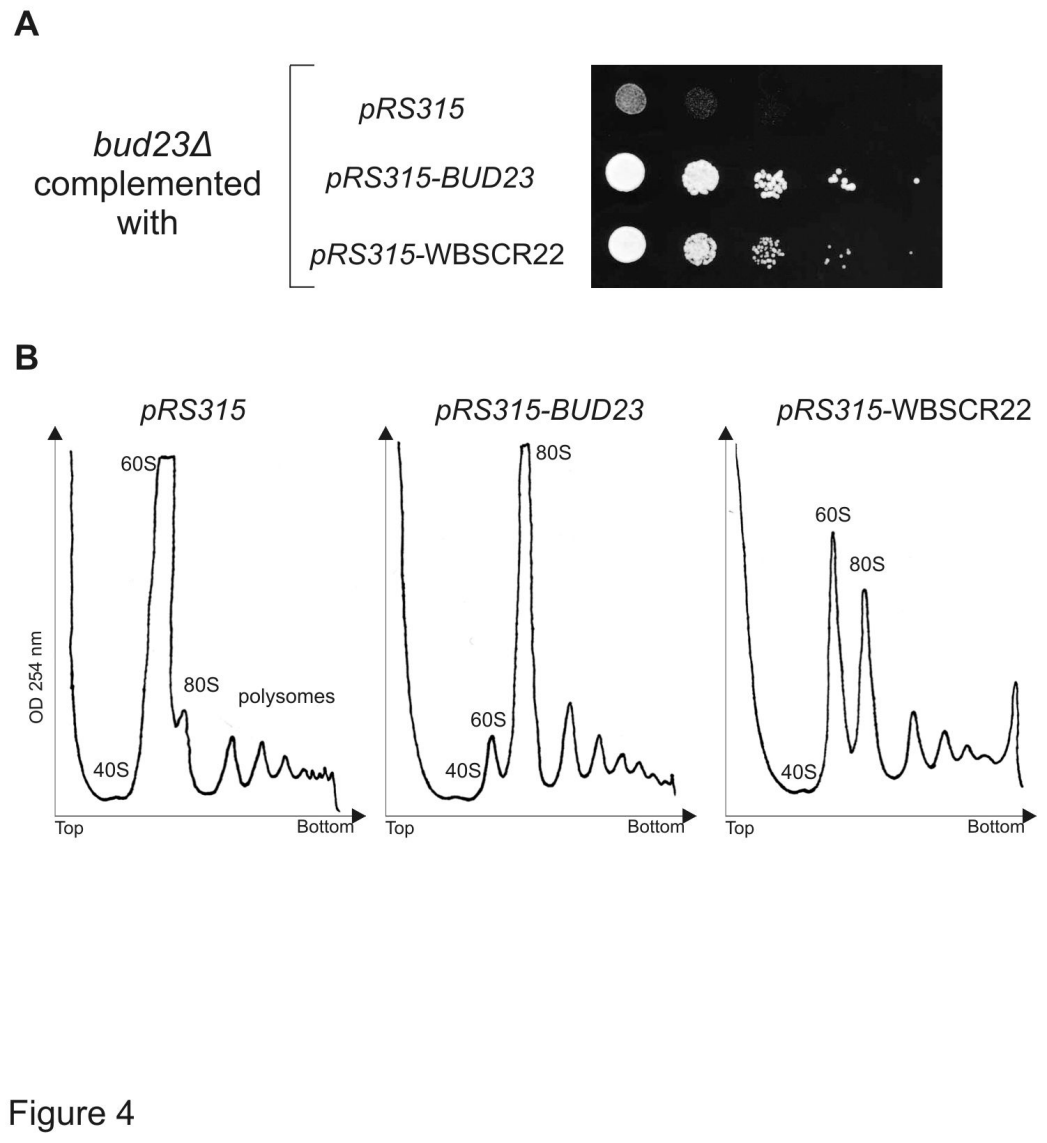
Complementation of yeast *bud23Δ* strain with human WBSCR22. (A) Growth dilution assays of *bud23Δ* carrying plasmids *pRS315*, *pRS315-BUD23* and *pRS315*-WBSCR22. Cultures were grown overnight and diluted to final optical density at OD_600_ 0.1, from which further 10-fold serial dilutions were spotted. Plates were incubated at 30°C for 3 days. (B) Polysome profiles of *bud23Δ* carrying plasmids *pRS315*, *pRS315-BUD23* and *pRS315*-WBSCR22. Cell lysates were centrifuged on 10-45% sucrose gradient. Absorbance at 254 nm was measured across the gradient, and the positions corresponding to the 40S, 60S and 80S ribosomal particles are indicated.

Our next goal was to find out whether the human WBSCR22 protein is a true homologue of yeast Bud23. For this, the WBSCR22 was cloned and expressed under the control of ADH promoter and tested for function. *Bud23Δ* yeast strain exhibits slow growth phenotype and defects in 40S subunit synthesis [9]. As shown in Figure 4A, the expression of yeast Bud23 as well as human WBSCR22 protein complemented the growth of *bud23* deletion mutant. However, the expression of human WBSCR22 protein complemented the growth of yeast *bud23*-null mutant only partially, WBSCR22-complemented cells grew always slower than *bud23Δ* cells expressing the yeast Bud23 protein itself. Analysis of polysome profiles confirmed the partial complementation of *bud23Δ* by *pRS315*-WBSCR22. The *bud23Δ* strain exhibits a strong 40S biogenesis defect resulting in a strong subunit imbalance with almost no free 40S, very high free 60S levels and reduced polysomes [9,15]. The expression of yeast Bud23 protein in *bud23Δ* cells suppressed the accumulation of free 60S and enhanced the formation of mature 80S particles and polysomes (Figure 4B). The expression of human WBSCR22 showed some subunit imbalance, however, this was less severe than in a *bud23Δ* strain transformed with empty vector (Figure 4B). In spite of these differences we suggest that the human WBSCR22 is a functional homologue of yeast Bud23 and these two proteins have similar functions.

### WBSCR22 does not stably associate with ribosomes

**Figure 5:**
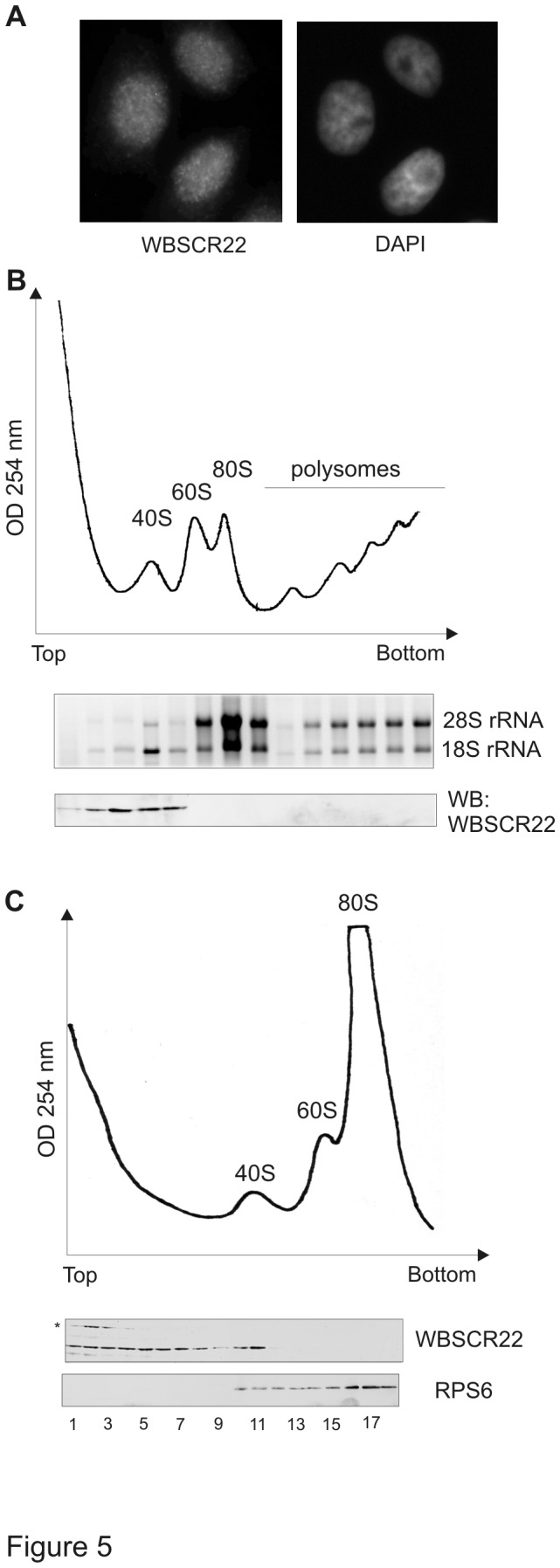
Localization of the WBSCR22 protein within the cell. (A) Subcellular localization of WBSCR22 by immunofluorescence analysis. The localization of endogenous protein in HeLa cells was analyzed by anti-WBSCR22 antibody. DAPI was used to stain the nucleus of the cell. (B) Total extracts of HeLa cells were centrifuged on 10-50% sucrose gradient at 27 000 rpm for 4 hours and fractionated. The 18S and 28S rRNAs were detected by ethidium bromide and the WBSCR22 protein was analyzed by western blotting using anti-WBSCR22 antibodies. (C) HeLa cell extracts were centrifuged on 10-50% sucrose gradient at 27 000 rpm for 13 hours and analyzed by western blotting.

Next we examined the localization and sedimentation profile of the WBSCR22 protein. By the immunofluorescence analysis, endogenous WBSCR22 protein showed predominantly a diffused signal throughout the cell nucleus (Figure 5A). In order to investigate whether WBSCR22 co-sediments with ribosomal particles, HeLa cell extract was centrifuged through a sucrose gradient followed by western blot analysis with WBSCR22-specific antibodies. As shown in Figure 5B, the WBSCR22 protein co-sedimented with pre-40S or 40S subunits containing 18S ribosomal RNA, but was not detected in fractions containing 60S subunits, assembled 80S particles or polysomes. The WBSCR22 protein signal was also detected in fractions close to the top of the gradient. Further analysis showed that WBSCR22 protein can be found in 40S fractions as well as in all fractions sedimenting slower than 40S particles (Figure 5C). Next we tried, but were not able to co-immunoprecipitate 18S rRNA with WBSCR22 from UV-cross-linked mammalian cells (data not shown). These data suggest that the WBSCR22 protein is not stably associated with ribosomes within the cell nucleus. We suggest that most of the WBSCR22 protein is localized freely or in complex with some other protein(s) within the nucleus of the cell. These data show that the WBSCR22 protein localization and distribution is different from its yeast counterpart Bud23, which has been shown to co-sediment with 90S ribosomal subunits and to localize to the nucleolar compartment of the cell [9,15,16].

### The WBSCR22 protein level is decreased in WBS patients’ lymphoblastoid cell lines

**Figure 6:**
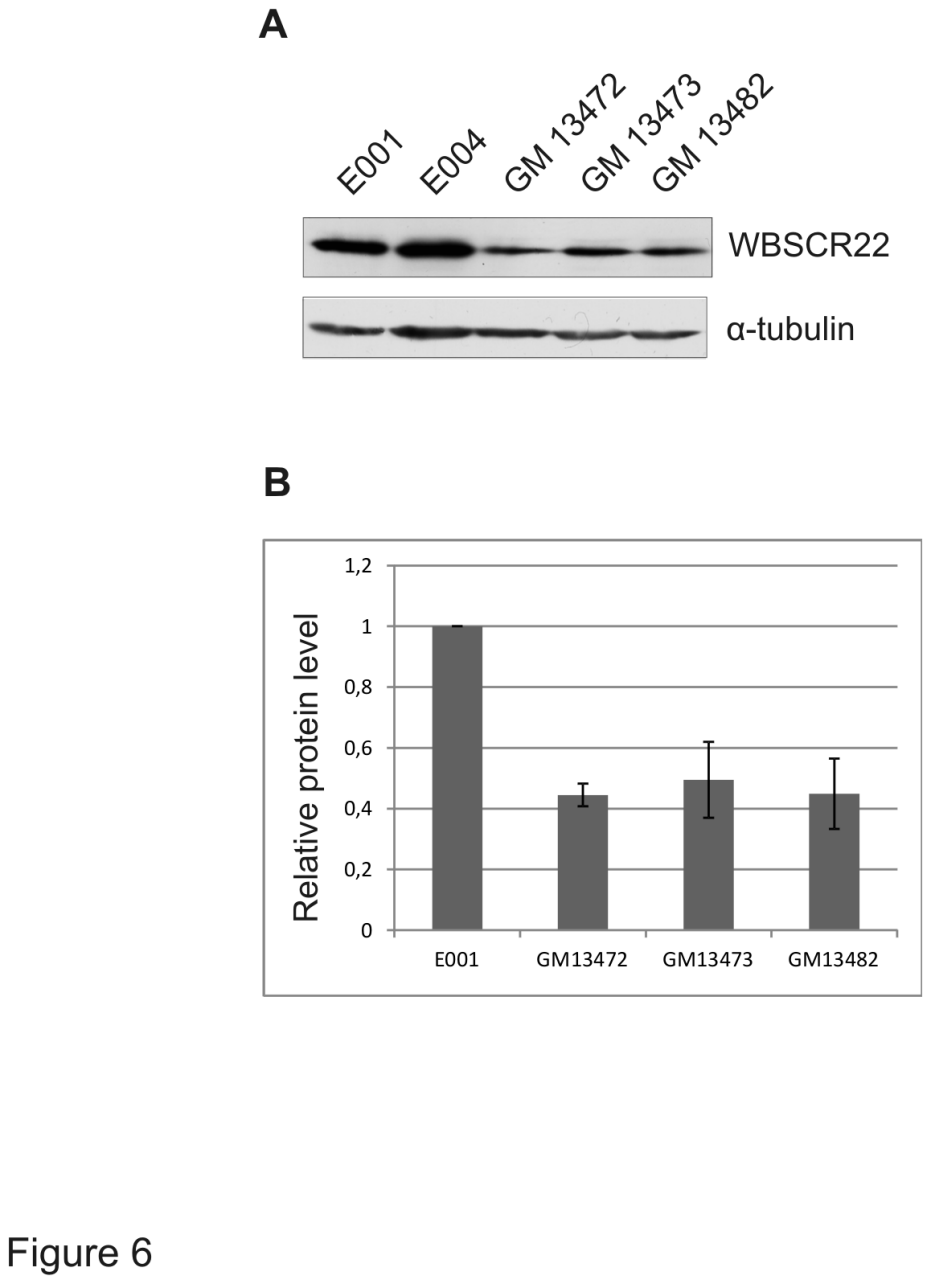
The WBSCR22 protein in WBS patient lymhoblastoid cell lines. (A) Extracts from wild-type (E001 and E004) and three WBS patient-derived lymphoblastoid cell lines (GM13472, GM13473, GM13482) were immunoblotted to visualise the indicated proteins. (B) Quantification of western blot analysis. Average of three independent experiments with standard deviations is shown. The P value is in all cases below 0.005 using Student’s t-test.

The WBSCR22 transcript encoded by the WBS control region is under-expressed in lymphoblastoid cell lines and fibroblasts derived from Williams-Beuren syndrome patients [8,17]. In order to test the WBSCR22 protein level in WBS patients, we performed immunoblot analysis of WBS lymphoblastoid cell lines used previously for transcription analysis [8]. Compared to healthy controls, the WBSCR22 protein level analyzed from three independent lymphoblastoid cell lines (GM13472, GM13473, GM13482) has decreased (Figure 6A). The protein level was reduced approximately 2.5-fold from wild-type level (Figure 6B) and correlated with the decrease of mRNA transcripts of these cell lines (data not shown). We also analyzed the ribosome profiles of lymphoblastoid cell lines, but could not detect differences between WBS patients and control cells (data not shown).

## Discussion

In this study, we show that the human WBSCR22 protein is involved in the processing of ribosomal RNA and synthesis of 40S subunits. Assembly of eukaryotic ribosomes is a complicated process that requires orchestrated co-action of different RNAs and proteins, including more than 150 non-ribosomal proteins, called trans-acting factors, to aid the assembly, maturation and intracellular transport of ribosomal subunits. These processes are relatively well-studied in yeast, but less is known in mammals. Here we show that the human WBSCR22 protein is a mammalian counterpart of yeast Bud23 involved in the assembly of 40S subunits. The WBSCR22 homologue in *Arabidopsis* , the RID2 protein, is also involved in pre-rRNA processing and is required for the establishment of a stable proliferative status [14]. We show that the down-regulation of WBSCR22 with siRNA decreases the amount of free 40S and changes the ratio of free 40S and 60S subunits in the cell. These data refer to defects in the processing and nuclear export pathway of pre-40S particles. Our finding is consistent with recent study by Wild et al. [18] where the requirement of WBSCR22 for biogenesis of 40S was proposed. The core features of ribosome assembly are conserved from yeast to human and a lot of proteins involved in processing and assembly of 40S particles are functionally conserved [11,12,19].

Two independent studies, our work and the previous study by Nakazawa et al. have both shown that the endogenous WBSCR22 protein is localized diffusely throughout the cell nucleus [4]. So, the human WBSCR22 and its homologs described so far have a different localization within the cell nucleus as both Bud23 and *Arabidopsis* RID2 proteins localize predominantly to the nucleolus of the cell [9,14]. Nucleolus has been suggested as a site of covalent RNA modifications and pre-40S assembly [20], pre-40S subunits are rapidly transported through the nucleoplasm and are not significantly detected there [21]. Most ribosome biogenesis factors associate with pre-ribosomal particles and Bud23 co-purifies with pre-40S particles [22] and co-sediments with 80/90S ribosomal subunits in yeast [16]. Our data show that the WBSCR22 protein does not form a stable complex with ribosomes, it localizes predominantly freely in the cell nucleus by immunofluorescence and co-sedimentation analysis. We suggest that human WBSCR22 and yeast Bud23 have similar, but probably not identical functions. The WBSCR22 protein complemented the slow growth of *bud23Δ* cells only partially. Knock-down of WBSCR22 had milder phenotype than deletion of *BUD23*, which is, however, also dispensable for budding yeast. It is possible that pre-rRNA processing has become more complex during the course of evolution and the impact of every particular factor has changed during this process. In addition, the functional redundancy may also be higher in mammalians than in yeast. The exact mechanism and the role of WBSCR22 in biogenesis of 40S particles need further investigations.

The WBSCR22 protein is a putative methyltransferase containing a SAM-binding motif typical of methyltransferases and its yeast ortholog Bud23 has been shown to methylate m^7^G1575 of 18S rRNA [9]. However, the methyltransferase activity of Bud23 is not required for its activity as Bud23 catalytic mutants unable to modify rRNA and G1575 mutation to A in 18S rRNA had little impact on cell growth [9]. Similarly, the Dim1 protein, which catalyzes the dimethylation of two adenine bases near the 3’-end of the 18S rRNA is an essential protein in yeast, but the processing of 20S pre-rRNA to 18S rRNA can still occur in the absence of dimethylation [23,24]. Furthermore, Emg1, a pseudouridine-N1-specific methyltransferase, catalytic activity is not required for ribosome biogenesis in yeast [25]. All these RNA methyltransferases have dual roles in assembly of 40S subunit and their involvement in ribosome assembly, rather than their methyltransferase activity, seems to render them important. We have no data about the methyltransferase activity of the WBSCR22 protein, but we suggest that the WBSCR22 protein itself is required for biogenesis of ribosome small subunits.

The WBSCR22 protein has a role in regulation of cell growth. Here, we show that the knock-down of WBSCR22 with siRNA results in slower growth of WBSCR22-depleted cells. This is consistent with previous findings using different cancer and transformed cell lines [4,5]. Ribosome synthesis is highly regulated and tightly coupled to cell growth. Actively dividing cells have a higher demand for ribosomes and various cancer cells show an increased expression of ribosomal and non-ribosomal factors involved in ribosome assembly ( [26] and references therein). The dysregulation of ribosome biogenesis has shown to be associated with tumor progression of breast adenocarcinoma MCF-7 cell line [27]. The ribosome assembly factor bystin is overexpressed in hepatocellular carcinoma and is required for cell growth and tumor development [28,29]. These findings suggest that WBSCR22 should be investigated further as potential therapeutic target in cancer cells.

The WBSCR22 protein is encoded by the *WBSCR22* gene located in WBS control region. This region contains 26-28 genes on chromosome 7q11, 23 and most of these genes show reduced expression levels if deleted hemizygously [8,17]. In the current study, we show that the WBSCR22 protein level has reduced approximately 2.5-fold in WBS patients compared to healthy controls. However, it is currently not known whether this decrease has any contribution to the development of disorder. We did not detect either slower growth of WBS patients’ lymphoblastoid cell lines or differences in ribosome profiles compared to healthy controls. Ribosome biogenesis is among the most fundamental molecular processes in the cells and in multicellular organisms, the process is linked to developmental regulation. In model organism *C. elegans*, inhibition of processing of 18S rRNA and subsequent formation of the 40S ribosomal subunits leads to various developmental abnormalities simultaneously [30]. The cell growth correlates closely with an increase in both protein synthesis and ribosome biogenesis, and regulators of ribosome biogenesis may play an important role in situations where rapid massive production of proteins or rapid proliferation is required. The WBSCR22 homologue in plant, *rid2* mutant inability to maintain active cell proliferation leads to defects in root growth [14]. The potential role of WBSCR22 alone and in concert with other proteins of WBS control region in the development of WBS syndrome needs further investigations.

## Materials and Methods

### Ethics statement

Two control human lymphoblastoid cell lines (E001 and E004) were established at the Institute of Technology, University of Tartu, from healthy volunteers, participating in a study of DNA copy-number variations in healthy individuals and patients with developmental anomalies in Estonia. This study was approved by the Ethics Review Committee on Human Research of the University of Tartu (reference number Protocol 197T-15) and appropriate written informed consent was obtained for each sample.

### Plasmids and siRNAs

To construct the WBSCR22 expression vector pQM-NTag-WB22, WBSCR22 cDNA was amplified from U2OS cells, obtained from American Type Culture Collection (ATCC) (number HTB-96) by PCR using WB22_R (5´-TGCTGAGGCGTGAGAATG-3´) and WB22_S6_F (5´-GCAAGTGCCTTTCCAGAA-3´) primers and cloned into pJET1/blunt vector (Thermo Scientific). HindIII site was generated at 5´ end of WBSCR22 by PCR using WB22Hind (5´-ATGAAGCTTATGGCGTCCCGCGGCCG-3´) primer and the product was cloned into the HindIII site of pQM-CMV-E2-N/A (Icosagen), in frame with the N-terminal epitope tag E2Tag.

Yeast vector pRS315-bud23 was made by amplifying bud23 ORF from genomic DNA using primers bud23F (5’-ATGGATCCATGTCACGTCCTGAGGAG) and bud23R (5’-ATGGTACCCTAGAACCTGTGTCTTC). The product was ligated into pRS315 vector as XbaI and OliI fragment. For pRS315-WBSCR22, the XbaI and SmaI fragment from pQM-Ntag-WB22 was ligated into XbaI and OliI sites of pRS315 vector. All the expression vectors were verified by sequencing.

Small interfering RNA (siRNA) duplex siWBSCR22 (5´-CGAGCAUUGGAGCUUCUUUAU-3´) was designed to knock down expression of human gene encoding WBSCR22 protein. As a control, negative siRNA (5´-CCCUGUCAGUAUUGAUAGAAA-3´) was used. siRNAs were purchased from Sigma-Aldrich.

### Cell culture and transfections

Human cervical carcinoma cells (HeLa) and human embryonic kidney cells (HEK293) were grown in Iscove’s Modified Dulbecco’s Media (IMDM) supplemented with 10% fetal calf serum (FCS), 100 U/ml penicillin and 100 µg/ml streptomycin. Cells were incubated at 37°C in 5% CO_2_ environment. Human lymphoblastoid cell lines from 3 individuals with WBS (GM13472, GM13472 and GM13482) [8] originating from the Coriell Institute for Medical Research Cell Repositories and from healthy controls (E001 and E004) were cultured in RPMI1640 medium supplemented with 10% FCS, 100 U/ml penicillin and 100 µg/ml streptomycin.

For siRNA knock-down, 100 µl of cell suspension (10^6^ cells) in Opti-MEM was mixed with 500 pmol of siRNA and transfected by electroporation in 4-mm cuvettes (Thermo, Fisher Scientific) using Bio-Rad GenePulser Xcell (settings square wave, 1000 V, 2 pulses, pulse length 0.5 ms). The cells were suspended in IMDM medium supplemented with 10% FCS and antibiotics

To follow the cell growth, HeLa cells were transfected with control and WBSCR22 siRNAs, aliquoted onto 60-mm culture dishes and analyzed 24, 48, 72, 96 and 120 hours after transfection. The cells were split after every 48 hours. At each time point, cells were collected with 3 mM EDTA in PBS and then resuspended in PBS. Then cells were stained with trypan blue and counted with Automated Cell Counter (Invitrogen).

For rescue experiments, 48 hours after siRNA electroporation the siWBSCR22 treated cells were transfected with 4 µg of empty vector or pQM-NTag-WB22 using Lipofectamine2000 (Invitrogen), following manufacturers protocol. 24 hours later cells were fractionated into nuclear and cytoplasmic compartments, RNA from each fraction was extracted and 18S rRNA processing was analysed by Northern blot.

### Immunofluorescence and immunoblotting

HeLa cells grown on glass coverslips were washed with PBS, fixed in 4% paraformaldehyde in PBS for 10 minutes at room temperature and permeabilized in 0,5% Triton X-100 in PBS for 5 minutes at room temperature. After three washing steps in PBS, cells were blocked for 30 minutes in PBS containing 0.25% bovine serum albumin (BSA). Cells were incubated with primary antibody against WBSCR22 (20 µg/ml, ab97911; Abcam) in 0.25% BSA in PBS for 1 hour at room temperature and then washed three times with PBS. The cells were then incubated for 1 hour with Alexa-488 conjugated secondary antibody (dilution 1:1000) (Invitrogen), washed three times in PBS and placed under coverslips with SlowFade® Gold antifade reagent with DAPI (Invitrogen). Analysis was performed with Nikon Eclipse TE2000-U fluorescence microscope.

For immunoblotting analysis, cells were lysed and proteins were separated by SDS-polyacrylamide gel electrophoresis and transferred by a semidry blotting method to a polyvinylidene difluoride (PVDF) membrane (Millipore Corp.). Membranes were incubated with anti-WBSCR22 (Santa Cruz Biotechnology), anti-Rps6 (sc74459, Santa Cruz Biotechnology) or anti-α-tubulin antibody (Sigma-Aldrich). Detection was performed using an ECL detection kit (GE Healthcare) following the manufacturer’s manual.

To study the expression level of WBSCR22 protein in control and in Williams-Beuren syndrome patient derived LCLs, cells were counted and equal number of cells was lysed. For immunoblotting analysis, the lysate of 100 000 cells was separated by SDS-PAGE, transferred to PVDF membrane and probed with antibodies against WBSCR22 and α-tubulin. Signals were detected by exposure to X-ray film or to RT ECL Imager (GE Healthcare). Western blot band intensities were analysed with ImageQuant TL software. The WBSCR22 protein level was normalized to tubulin level and the average of three independent experiments are represented.

### Cell fractionation and RNA extraction

72 hours after transfection with siRNAs, HeLa or HEK293 cells were collected, washed with PBS and hypotonic buffer A (10m M Tris-HCl, pH 7.5; 2 mM MgCl_2_; 10 mM KCl). Then cells were suspended in 300 µl of buffer A containing 0.5 mM DTT and after 20 minutes of incubation on ice mechanical disruption was performed with 10 strokes in Dounce homogenizer. After centrifugation at 1000 g for 10 minutes at 4°C, the supernatant (cytoplasmic fraction) was collected. The pellet containing nuclei was washed with buffer A and resuspended in 300 µl of nuclear lysis buffer B (25 mM Tris-HCl, pH 7.5; 100 mM KCl; 1 mM DTT; 0.5% Triton X-100; 2 mM EDTA). After 10 minutes of incubation on ice, the suspension was centrifuged at 1500 g for 15 minutes at 4°C and the supernatant (nuclear fraction) was collected. RNA from whole cells, from cytoplasm and from nucleus was extracted with Trizol reagent (Invitrogen) following manufacturers’ instructions.

### RNA analysis

18S rRNA processing was analysed by Northern blot. 3 µg of total and nuclear RNA and 6 µg of cytoplasmic RNA were separated electrophoretically on 1% agarose gel in MOPS buffer (20 mM MOPS, 2 mM sodium acetate, 1 mM EDTA pH 8,0) containing 6% formaldehyde and blotted onto Hybond positively charged nylon membrane (GE Healthcare). The membrane was washed in 6xSSC buffer for 15 minutes at room temperature, dried and fixed with UV light. The membrane was prehybridized in 20 ml buffer containing 6xSSC, 5xDenhardt, 0.5% SDS and 100 µg/ml carrier sperm DNA for 1 hour at 45°C. Blots were hybridized with [^32^P]-labelled 5’ ITS1 oligonucleotide probe (5´-CCTCGCCCTCCGGGCTCCGTTAATTGATC) at 45°C overnight. After hybridization, membrane was washed in washing buffer I (2xSSC, 0.1% SDS), washing buffer II (1xSSC, 0.1% SDS) and washing buffer III (0.1xSSC, 0.1% SDS), 2x10 minutes each. Labeled RNA signals were detected by exposure to X-ray film or Typhoon Phosphoimager (GE Healthcare) and analysed using ImageQuant TL software. Quantification of 18/28S was made from agarose gels using ethidium bromide as a dye with Typhoon Phosphoimager and ImageQuant TL software.

### Analysis of ribosomes by sucrose density gradient centrifugation

72 hours after transfection with siRNAs, HeLa cells were treated with 50 µg/ml cycloheximide (Sigma) for 10 minutes. To prepare ribosomes from whole cells, about 10^7^ HeLa cells were collected, washed in PBS containing 50 µg/ml cycloheximide and lysed for 10 minutes on ice in 500 µl lysis buffer (20 mM Tris-HCl, pH 7.5, 3 mM MgCl_2_, 150 mM KCl, 1 mM DTT, 0.5% Triton X-100, 50 µg/ml cycloheximide). The lysate was cleared by centrifugation at 13 000 rpm for 10 minutes at 4°C and supernatant was preserved for analysis. Alternatively, 1.5-2x10^7^ of cells were fractionated into cytoplasmic fraction as described above, except for 50 µg/ml cycloheximide was added to PBS and buffer A. Total and cytoplasmic extracts were layered on a 10-50% sucrose gradient (10-50% sucrose (w/V), 20 mM Tris-HCl, pH 7.5, 100 mM KCl, 3 mM MgCl_2_) and centrifuged for 4 hours at 27 000 rpm in a SW41 rotor (Beckman). The gradient was analysed at OD_254_ nm.

### Protein and RNA analysis from sucrose gradients

To study the sedimentation of WBSCR22 protein, HeLa cell lysate was layered on a 10-50% sucrose gradient and centrifuged at 27 000 rpm for 4 hours (Figure 5B) or 13 hours (Figure 5C). Gradients were fractionated, proteins were precipitated with 10% trichloroacetic acid, separated by SDS-polyacrylamide gel electrophoresis and analyzed by western blot using antibodies against WBSCR22 and Rps6. RNA was extracted with phenol/chloroform, precipitated with isopropanol and analysed by 1.2% agarose gel.

### Yeast experiments

For complementation assay, plasmids pRS315, pRS315-BUD23 and pRS315-WBSCR22 were transformed into *bud23Δ* (MATa *bud23::kanMX his3Δ1 leu2Δ0 ura3Δ0 met15Δ0*) yeast strain AJY2161 [9] by standard method. Yeast strains carrying pRS315 derived plasmids were cultured in synthetic complete media minus leucine supplemented with 2% glycose at 30°C. For growth dilution assays, cultures were grown overnight and diluted to final optical density at OD_600_ 0.1, from which further 10-fold serial dilutions were prepared and spotted (5 µl) onto -LEU plates. For polysome analysis, cultures were grown to optical density OD_600_ 0.5-0.8, cycloheximide was added to final concentration of 0.1 mg/ml, and cells were collected by centrifugation. All the steps were carried out on ice. Cell pellets were washed with buffer (10 mM Tris-HCl; pH 7.5, 100 mM KCl, 10 mM MgCl_2_, 1 mM DTT, 0.1 mg/ml cycloheximide), resuspended in 600 µl of the same buffer, and broken by vortexing in the presence of glass beads. The extract was centrifuged for 10 min at 16 000 g at 4°C, and the supernatant was recovered. Fifteen OD_260_ units were loaded onto a 10-45% sucrose gradient and centrifuged for 15 hours at 17 000 rpm in a SW28 rotor (Beckman). The gradient was analysed at OD_254_ nm.
